# Knowledge of HIV/AIDS and use of mandatory premarital HIV testing as a prerequisite for marriages among religious leaders in Sokoto, North Western Nigeria

**Published:** 2012-02-16

**Authors:** Sambo Adamu Umar, Oche Mansur Oche

**Affiliations:** 1 Usmanu Danfodiyo University Teaching Hospital, Sokoto, Nigeria

**Keywords:** Knowledge, HIV/AIDS, premarital, HIV testing, religious leaders, Nigeria

## Abstract

**Background:**

In Sub-Saharan Africa, an estimated 1.8 million became infected with the HIV in 2009 and Nigeria currently has about 3.4 million people living with HIV. Measures put in place by religious organizations to combat HIV/AIDS in Nigeria include mandatory premarital HIV testing. The knowledge of HIV/AIDS amongst religious leaders in Nigeria has not been sufficiently explored . In this study, we assessed the knowledge of HIV/AIDS amongst religious leaders in Sokoto and if they routinely demand for mandatory premarital HIV testing for all intending couples.

**Methods:**

This was a descriptive cross-sectional study involving 158 religious leaders (30 Christians and 128 Muslims) who officiate or assist during marriages. Data was collected using interviewer and self administered questionnaire which sought such information as biodata, knowledge of HIV/AIDS , speaking to congregation about AIDS and using Premarital HIV status as a pre-requisite for contracting marriages. Data was entered into and analysed using Epi-info computer soft ware program. Level of statistical significance was put at P=0.05.

**Results:**

The ages of the respondents ranged from 35 to 78 years with a mean age of 26.3 ± 20.3years. Forty nine percent of the respondents had adequate knowledge of HIV/AIDS with more Christian clerics compared to Muslim Clerics having better knowledge of HIV/AIDS (P<0.0001). All the Christian clerics opined that they would insist on mandatory premarital HIV testing for their subjects before joining them in marriages.

**Conclusion:**

The results of the study have shown that most of the religious leaders lacked adequate knowledge of HIV/AIDS and the use of mandatory premarital HIV testing is yet to be adopted by the Muslim clerics. Awareness campaigns should be intensified for the religious leaders to improve their knowledge of HIV/AIDS.

## Background

Acquired Immune Deficiency syndrome (AIDS) has become the leading cause of premature death in Sub-Saharan Africa and the fourth largest killer worldwide [1]. In Sub-saharan Africa, where the majority of new HIV infections continue to occur, an estimated 1.8 million people became infected in 2009, considerably lower than the estimated 2.2 million people in sub-saharan Africa newly infected with HIV in 2001. Nigeria currently has about 3.4 million people living with HIV [2].

In most people's minds, the HIV virus is tied to behaviour that is religiously condemned as it has been equated with “a curse” and those who live with it have been viewed as “sinners” [3]. The response to HIV/AIDS in Nigeria has not been limited to government or government agencies alone. Other sectors and nongovernmental organisations including religious bodies are also involved. In the early days of the epidemic, the prevailing view among religious leaders was that AIDS was God sent as a punishment for man's iniquities [4]. However this widely held view has since changed with the coming on board of many religious bodies in Nigeria leading the fight against HIV/AIDS.

Since the advent of the AIDS pandemic, the disease has exposed religious leaders to be as ignorant as those they lead. In most countries, religious leaders have been blamed for their foot dragging, reluctance to speak openly and their general passiveness as the epidemic swept through their congregation and countries. Moreover, religious organizations and their leaders have most often than not, been known for their emphasis on abstinence and mutual fidelity. According to McCain, AIDS is affecting the religious community as many people suffering from AIDS are religious people who are members of churches and mosques [5]. Religious leaders contribute to the shaping of social values and norms, and can influence public attitudes and national policies related to the epidemic [3]. The role of religious leaders has gone beyond prayers, fasting and other religious obligations and juristic regulations to include other social obligations such as mobilising people for various medical, social, cultural and religious activities.

Measures put in place by religious organizations to combat HIV/AIDS have elicited some controversies and one particular response which has attracted a lot of controversies is mandatory pre-marital HIV testing. Mandatory premarital HIV testing refers to the requirement of an HIV test as a condition for entering into marriage. The practice of mandatory pre-marital HIV testing which originated from the states of Louisiana and Illinois in the United States has also been documented in Nigeria, Democratic Republic of Congo (DRC), Ghana, Burundi and Uganda [6]. National governments in Bahrain, Guinea, the United Arab Emirates and Saudi Arabia have enacted laws and policies mandating premarital HIV testing [7]

In Nigeria, the Anglican church considers as unacceptable, the AIDS pandemic and has therefore joined other denominations in demanding that church members intending to wed undergo mandatory premarital HIV tests to qualify to marry in the church. This, the church said was to contain the spread of the disease [8]. Other denominations in Nigeria especially the Pentecostal Churches, have since adopted this stance [8]. Premarital testing has been found to reduce HIV infection rates by “containing” infection within the population of people living with HIV and also encourages couples to practice “moral behavior” abstinence before marriage and fidelity after marriage which in turn will slow the spread of HIV infection[6].

The Muslim community in Nigeria has done very little in this direction. However churches in Africa under the aegis of All African conference of churches (AACC) promised to close the gaps caused by denominationalism and develop common policies to end the culture of silence which promotes stigmatization and initiate effective responses to prevention, care and counselling [9]. However in Nigeria, studies conducted to determine the prevalence of HIV infection among intending couples referred from religious organizations for pre-marital HIV testing showed a relatively high HIV prevalence among intending couples. This may seem to justify the rationale for the institution of mandatory pre-marital HIV testing by some religious organizations [10,11].

Awareness, knowledge and the capacity to act can add the much required value to efforts aimed at reducing the scourge of HIV/AIDS in our society. Studies from Jordan showed that the role of religious leaders in disseminating AIDS related information was reported to be minimal [12,13]. In a study in Uganda among Imams, it was observed that 86% of the respondents had correct knowledge of the mode of transmission of HIV [14]. In the same study, only 15% of the Imams would advocate the use of condoms as a means of prevention of HIV infection. In Jimma zone of Ethiopia, it was observed that majority of the religious leaders spoke openly to their congregations about HIV/AIDS with only a few reluctant to discuss this topic in churches and mosques; however, sensitive subjects such as sexual transmission of HIV and the use of condoms proved difficult to discuss [15], even though there is no scientific evidence that the more people talk about condoms, the more promiscuous people become.

For the increasing upsurge in the prevalence of HIV/AIDS to be contained, there is the need to come up with programs that will provide AIDS education and awareness among religious leaders. Literature search has shown that no similar studies have been carried out in this part of Nigeria to assess the knowledge of religious leaders about HIV/AIDS and their use of mandatory premarital testing for HIV in marriages. The knowledge of HIV/AIDS amongst religious leaders particularly in Sokoto and Nigeria in general has not been sufficiently explored; the study therefore assessed the knowledge of HIV/AIDS amongst religious leaders in Sokoto metropolis and also to determine if they routinely asked would-be couples in their congregations to undertake mandatory premarital HIV tests before joining them in marriage.

## Methods

Approval was obtained from both the Christian Association of Nigeria, Sokoto state and the Arabic and Islamic Board and Sokoto state ethical clearance committee.

This descriptive cross sectional study was carried out in Sokoto metropolis of Sokoto State in North Western Nigeria from September to November 2010 and was aimed at assessing the knowledge of religious leaders with respect to HIV/AIDS and if they routinely asked intending couples to undergo mandatory premarital HIV testing. The study population comprised of all religious leaders in Sokoto metropolis made up of Reverend Fathers, Pastors, Islamic and Christian religious teachers, Islamic preachers, Imams and deputy Imams who officiate or assist in wedding ceremonies in their denominations not later than one year ago(Eligibility criterion). A line list of all the clergy practicing in Sokoto metropolis was obtained from the Christian Association of Nigeria and the Arabic and Islamic Board in Sokoto comprising of 59 and 251 Christian and Islamic clergy men. A sample size of 167 was calculated with proportionate allocation of samples to each group. A total of 30 Christian and 128 Islamic clergymen were randomly selected and agreed to participate in the study.

Verbal informed consent of the respondents was obtained before the commencement of the study and participants were given options to opt out when they desired. Six research assistants trained on the objectives of the study and the study instrument, collected data using interviewer and self administered questionnaire which sought such information as age, length of practice as a clergy, knowledge of HIV/AIDS, speaking to congregation about AIDS and using Premarital HIV status as a pre-requisite for contracting marriages. The questionnaires were translated to Hausa (the predominant local language in the study area) by a Hausa scholar and back translated to English by another scholar.

The questionnaires were sorted out for completeness and accuracy. Knowledge of HIV/AIDS was assessed through twelve questions. Each correct answer to the questions attracted one mark with no marks awarded to wrong answers. Scores were then converted to percentages with Scores less than 50 and equal to or greater than 50% adjudged inadequate and adequate knowledge respectively. Data was entered into and analysed using EPI Info 3.3.2 software computer program and Graph pad Instat with level of significance of variables set at P = 0.05.

## Results

Of the 167 questionnaires sent out, 158 were correctly filled and returned (94.6% response rate). A total of 158 respondents were recruited into the study comprising of 128 (81%) Islamic and 30 (19%) Christian leaders. The ages of the respondents ranged from 35 to 78 years with 32% of them between 41 and 50 years of age and a mean age of 47 ± 3.1years (Table 1). A total of 42 (27%) of the respondents had tertiary education, 21 (13%) primary education while 44 (28%) had only Quranic education (Table 2). Out of 158 respondents, almost half 78 (49%) had adequate knowledge of the cause, mode of transmission and prevention of HIV/AIDS, while 80(51%) had inadequate knowledge of the infection. Of the 78 respondents who had adequate knowledge of HIV/AIDS, 30 were Christian clerics with all of them having formal education while 48 were Muslim clerics with 33 of them having formal education ( P<0.0001). There was a statistically significant relationship between the educational level of the respondents and their knowledge of HIV as those with formal educational had better knowledge of the infection and its mode of transmission. ( P<0.0001) (Table 3). Similarly, the relationship between age and the knowledge of HIV/AIDS was found to be statistically significant (P=0.0003) (Table 4). The commonest source of information on the infection was Radio and Television (45%) (Table 5). Majority 101 (64%) of the respondents would include total abstinence and mutual fidelity in sermons to their congregations while only 10 (6%) of the Christian clerics would talk on the use of condoms by their subjects (Table 6).

**Table 1:** Age distribution of respondents

Age (years)	No (%)
30-40	45 (29)
41-50	50 (32)
51-60	38 (24)
61-70	11 ( 7)
71-80	3 (2)
NR	11 ( 7)
**Total**	**158 (100)**

Mean age = 47±3.1 years

**Table 2:** Educational status of respondents

Educational level	Muslims	Christians

	No (%)	No (%)
Quranic only	95 (74.2)	0 (0)
Primary	21 (16.4)	0 (0)
Secondary	12 ( 9.4)	9 (30.0)
Tertiary	0 (0)	21(70.0)
**Total**	**128 (100)**	**30(100)**

**Table 3:** Relationship between educational status of respondents and knowledge of HIV/AIDS

Educational status	Adequate Knowledge of HIV/AIDS		Total
	
	Christian Clerics	Muslim Clerics	
Formal	30	33	63
Non formal	0	15	15
**Total**	**30**	**48**	**78**

X^2^_c_ =9.68, df=1, P=0.0019 (Significant)

**Table 4:** Relationship between age and knowledge of HIV/AIDS

Age (years)	Knowledge of HIV/AIDS		Total
	
	Adequate	Inadequate	
≤40	33	12	45
>40	45	68	113
**Total**	**78**	**80**	**158**

X^2^=13.2; df= 1;P=0.0003 (significant)

**Table 5:** Respondents’ source of information on HIV/AIDS

Source of information	No (%)
Media (Radio/TV)	71 (45)
HIV campaigns /lectures	26 (17)
Hospitals/Health workers	48 (30)
NR	13 (8)
**Total**	**158(100)**

NR= No response

**Table 6:** Contents of sermon to congregation about HIV/AIDS

Contents of sermon	No (%)
Mode of transmission of HIV	65 (41)
AIDS is a white man's propaganda	56 (35)
Total abstinence/mutual fidelity	101(64)
Advocating use of condoms for prevention	10(6)

* Multiple answers allowed

Most of the respondents 93, (59%) were of the opinion that abstinence and mutual fidelity were the only ways to curb the menace of HIV/AIDS while only 10 (6%), all Christian clerics, would advocate the use of condoms for prevention (Table 7). All the Christian clerics opined that they would insist on couples undergoing mandatory premarital HIV testing before joining them in wedlock as had been the practice, while all the Muslim clerics would not use premarital HIV status as a pre-requisite for marriages. The only reason given by the Christian clerics for insisting on HIV tests was to protect an uninfected partner from contracting the infection, thus reducing the spread of the disease and to discourage the practice of premarital sex amongst their congregation. Out of the 30 Christian respondents, ten were aware of couples who tested positive after the tests and were refused marriage in the church.

**Table 7:** Respondents’ opinion on prevention of HIV/AIDS

Prevention of HIV/AIDS	No (%)
Use of condoms	10 (6)
Abstinence/mutual fidelity	93(59)
Obey God's laws	55 (35)
**Total**	**158 (100)**

Some,34 ( 22%) of the Muslim clerics were of the opinion that they can only insist on mandatory premarital HIV test if they got an instruction from the highest Islamic body as they do not have the right to stop intending couples from getting married to themselves and moreover, they insisted that God had always provided a remedy for any disease discovered on earth.

## Discussion

This study revealed inadequate knowledge about HIV/AIDS among the religious leaders with only about half, 78 (49%) of the respondents having adequate knowledge of HIV/AIDS . This figure is low when compared to over 80% knowledge level obtained in the family AIDS education and prevention through Imams in Uganda [14]. The low level of knowledge obtained in this study could be attributed to the fact that discussions on issues of sexuality among religious leaders are often misconstrued by their congregations. However, the Christian respondents with formal education tended to have better knowledge of HIV, ( P< 0.0001) compared to their Muslim counterparts. This may not be unrelated to the fact that all the Christian clergymen had formal education, and had all along been speaking on the subject of AIDS hence their insistence on mandatory premarital HIV testing before marriage in the church. A base line research conducted in late 1994 among church leaders and church going youths in Kenya, revealed that majority of the church leaders (97%) recognised AIDS as a major problem in their communities, but most (67%) did not know how to address the problem [16]. If the current state of HIV/AIDS in the country must be nipped in the bud, then discussions on sexuality and how individuals could prepare themselves to make informed, meaningful and healthy choices that reflect who they are must be addressed by religious leaders in their daily sermons. . The media (Radio and Television) were the most common means of information about HIV/AIDS and this is consistent with findings from a similar study [14].

Majority of the respondents (64%) volunteered that during sermons to their congregations, they would only talk on abstinence and observance of mutual fidelity. This is in consonance with the findings from Ethiopia and Egypt where religious leaders agreed they had a lot to contribute in tackling the problems and would speak openly to their congregations provided they have all information concerning the HIV infection [15,17].

There was a general objection to the mentioning of condoms during sermons as only 6% of the religious leaders (Christians) agreed to talk on condoms and its use as a means of preventing HIV/AIDS to their congregation. This low figure may not be unconnected with the general belief that advocating the use of condoms by religious leaders would amount to an open cheque for promiscuity. The figure obtained in this study is low when compared with the figure obtained in Kenya where 42% of church leaders said they would likely counsel on the use of condoms but only when one marriage partner is unfaithful [18]. The recommendation of the use of condoms as a means of prevention has not received the blessings of religious leaders all over the world as they believed that condoms may facilitate the transgression of abstinence and the degradation of self discipline. In Malaysia, although knowledge about condom use was considered useful, several religious leaders said that they would not speak in public about condom use in non-marital sex as this was “haram” (forbidden) in Islam [19]. Switzerland promoted condom use and now the result is that many youngsters choose to postpone sexual involvement. Similarly, Uganda did the same by allowing people to talk about all the methods to prevent AIDS. Now increased abstinence among young people is obvious and there is no scientific evidence that the more you talk about condoms, the more people become promiscuous [20]. According to the Salvation Army church of Trinidad and Tobago ( T& T ), “sex before marriage is not acceptable in the Christian church, but being practical in this day and age, better use a condom than get a disease” [21].

Findings from our study showed that 30 (19%) of the Christian religious leaders justified their insistence on HIV testing among would be couples as the surest way of protecting the innocent partner from being infected with the HIV virus. Umeora et al in their study recommended that the screening of intending couples could play an important role in HIV detection in the general population [22]. This is hinged on the premise that if one partner is found to be HIV positive, the marriage is discontinued and would have gone a long way to detect those with the infection early. A study in Ghana showed that some religious leaders believed that their insistence on mandatory pre-marital HIV testing policy was their genuine way of protecting those who are HIV negative from becoming infected [23]. In Malaysia, the possibility of making the HIV test a compulsory pre-requisite for couples is receiving a boost following an increase in infection rates among Muslim youths in the country; the country's AIDS council noted that “In comparison with other religions, it is worrying to note that Islam seems to be left behind in most nations’ confrontation with HIV/AID” [24].

The religious leaders in our study who would not insist on HIV tests for intending couples believed that “God gives life and at the time God made diseases, a cure was provided for them”. Their not acting at the right time could spell doom for the congregation which they lead. The religious bodies and their leaders need to take a proactive stance if the scourge of the HIV/AIDS is to be nipped in the bud. We may chose to arrest the situation now or watch as the disease sweeps through and decimate our congregations.

## Conclusion

The results of this study showed that despite the scourge of the HIV/AIDS in the country, there is a wide gap in the knowledge of the religious leaders about this infection. There were discordant views with regards the use of mandatory premarital HIV testing as all the Muslim clerics would not insist on prospective couples undergoing the test before joining them in wedlock. This has serious implications for the continued use of mandatory pre-marital HIV testing by religious organizations as a strategy to curtail the spread of HIV infection. Given proper information and training, the religious leaders can become strong allies in HIV/AIDS prevention and control in view of their influence and acceptance among the people they lead. Also religious organizations can serve as useful channels for disseminating messages since congregations do have reverence for places of worship and for religious leaders.

### Recommendations

Increasing awareness and knowledge of the religious leaders especially the Muslim clerics about HIV/AIDS will erase all the misconceptions they have regarding the infection and hence embrace the policy of premarital HIV testing. Also,the religious bodies and their leaders should be incorporated into the state and national AIDS control agencies where culturally sensitive and sustainable programmes are designed and adopted for different communities. The Nigerian government need to borrow a leaf from countries that have made premarital HIV testing mandatory as a way of detecting couples who are HIV positive, thus reducing the spread of the infection.

### Limitations of the study

The study area is a predominantly Muslim state with a small proportion of Christians who are mainly non indigenous tribes. The selection of 30 Christian clergy may not be sufficient enough to establish possible relationships with some selected variables
